# Number of Teeth According to Hand Strength in Adults: A Cross-Sectional Study of 11,499 South Korean Adults

**DOI:** 10.3390/medicina59081373

**Published:** 2023-07-27

**Authors:** So-Yeong Kim, Sun-A Lim

**Affiliations:** 1 Department of Preventive Medicine, Chosun University Medical School, Gwangju 61452, Republic of Korea; soyeong4897@chosun.ac.kr; 2 Department of Dental Hygiene, Songwon University, Gwangju 61756, Republic of Korea

**Keywords:** hand strength, KNHANES, number of teeth, oral medicine

## Abstract

*Background and Objectives*: Grip strength is a measure of general health and is associated with oral health. This study aimed to investigate the relationship between the number of teeth and grip strength in Korean adults. *Methods and Materials*: We recruited adult participants who underwent oral examinations in the Korean National Health and Nutrition Examination Survey (KNHANES) between 2016–2018. When performing the KNHANES oral examination, an experienced dentist performed it according to the oral examination guidelines and recorded the results. A total of 24,269 participants from the 2016–2018 KNHANES were included in this study. Of these, only 16,489 participants underwent oral screening. A total of 3209 were non-adult children and adolescents, and 1781 did not respond to the grip strength test; those who did not respond to the main independent confounder were excluded. The relationships between grip strength, general characteristics, general health, oral health, and the number of remaining teeth were analyzed. There were 11,499 subjects in total in this study, with 4839 males and 6660 females. The age groups were 19–65 years of age, with 8387 subjects, and 65 years and older, with 3112 subjects, and the number of remaining teeth was 0–9 for 936 subjects, 10–19 for 1015 subjects, and 20–28 for 9548 subjects. *Results*: The probability that the higher the grip strength, the higher the residual number of gingiva was estimated by multinomial logistic regression analysis using complex sampling. The higher the grip strength, the higher the probability of having 20–28 teeth remaining (adjusted odds ratio, 1.59; 95% confidence interval, 1.19–2.13). *Conclusions*: Maintaining general health is related to maintaining teeth; the higher the grip strength, the higher the number of remaining teeth.

## 1. Introduction

Physical ability, a concept called bodily function, describes a person’s ability to perform bodily tasks in daily living. Measures of objective indicators of performance, such as grip strength, walking speed, standing, and balance, characterize physical fitness. There is also growing evidence that it serves as an indicator of health currently and in the future [1]. In particular, grip strength can be used as a measure of sarcopenia and is used as a measure to predict accelerated decline in activities of daily living, disability, and cognitive decline that contribute to dependence [2].

Regression of grip strength can cause death, due to physical health problems and cognitive decline; when grip strength is weak, dementia occurs and is related to increased mortality, due to cardiovascular and respiratory diseases [3]. In addition, low grip strength is associated with chronic diseases [4,5,6]; the higher the grip strength, the higher the health-related quality of life. The improvement of muscle strength can improve the health-related quality of life [7].

Oral health is closely related to individual physical abilities, as well as systemic and chronic diseases [8]. Previous studies have confirmed that tooth loss is an important predictor of mortality as an oral health indicator [9]. As a health outcome indicator that deteriorates with age, the number of teeth is used as a final indicator of oral and general health [10]. Moreover, tooth loss in adults can affect muscle conditions by reducing diet quality and the intake of most nutrients [11]. Accordingly, a relationship between tooth loss and handgrip strength can be assumed [12].

Most previous studies focused on the elderly population. In the case of the elderly, it was found that the faster the walking speed, the greater the muscle strength and the greater the number of remaining teeth [13,14]. It was not possible to objectively evaluate the number of teeth by using a self-report form [14]. There was also a study that investigated the relationship between the number of remaining teeth and grip strength in adults [3]. However, socioeconomic status and health behavioral factors were not completely adjusted as confounding factors [15]. Accordingly, in a representative general population sample, an association between the number of natural teeth remaining and hand strength after adjusting for potential confounding factors is needed.

Therefore, in this study, we investigated the relationship between the number of remaining teeth and grip strength using the representative Korean National Health and Nutrition Examination Survey data (KNHANES, 2016–2018) and prepared basic data to prevent a decline in general health or the number of teeth.

## 2. Materials and Methods

This study was reported in accordance with the Strengthening the Reporting of Observational Studies in Epidemiology guidelines (STROBE) [16].

### 2.1. Research Data

The National Health and Nutrition Examination Survey is a reliable data representative of the national health status and is a national cross-sectional survey that investigates the overall health status, chronic diseases, food and nutrient intakes, and oral health status of the people of Korea. The stratified cluster sampling used by the Korea National Health and Nutrition Examination Survey was used to select a nationally representative sample. In KNHANES, the overall health and oral health statuses of the subjects were collected through health surveys and health examinations at the mobile examination center. A total of 31,689 people aged 1 year or older were selected as subjects of the 7th National Health and Nutrition Examination Survey, of which only 24,269 people (76.6% of the total participants) participated. The KNHANES protocol was approved by the Korea Centers for Disease Control and Prevention Review Board (2018-01-03-P-A), and informed written consent was obtained from each participant prior to conducting the surveys and tests. Oral examination was jointly conducted by a dentist belonging to the Korea Centers for Disease Control and Prevention and a public health doctor. The oral examinations of the 7th National Health and Nutrition Examination Survey were conducted in small groups, due to the limited number of public health doctors in Korea.

### 2.2. Exclusion Criteria

**Figure 1:**
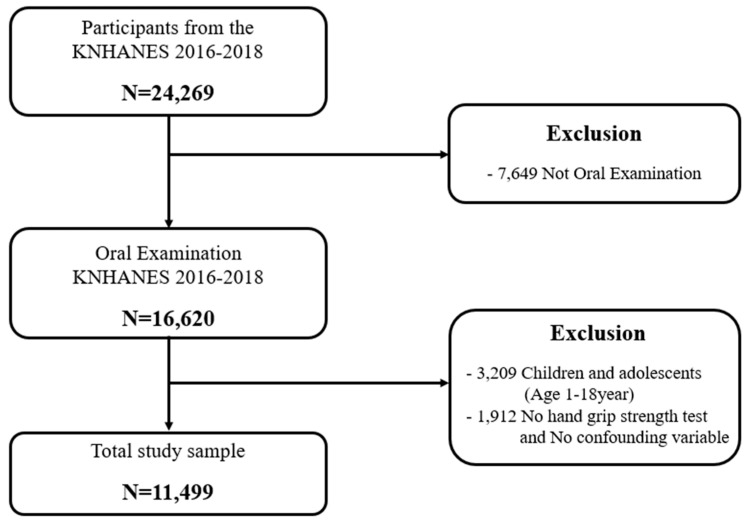
Participant flow diagram for final analysis.

A total of 24,269 participants from the 2016–2018 KNHANES were included in this study. Of these, only 16,489 participants underwent oral screening. Among the 16,489 patients, 3209 were non-adult children and adolescents, and 1781 did not respond to the grip strength test; those who did not respond to the main independent confounder were excluded. The final sample of this study consisted of 11,499 participants (Figure 1).

### 2.3. Evaluation of the Number of Existing Permanent Teeth

When performing the KNHANES oral examination, an experienced dentist performed it according to the oral examination guidelines and recorded the results. The number of existing permanent teeth was recorded, excluding missing, impacted teeth, implanted teeth (tooth transplant, implant, etc.), and third molars. The number of teeth was classified into three groups: 0–9, 10–19, and 20–28 teeth. The Korean oral health index and severe tooth loss index of previous studies were used as the criteria for this classification [17,18].

### 2.4. HandGrip Strength Evaluation

Participants’ grip strengths were measured using a digital grip dynamometer (Digital grip strength dynamometer, T.K.K 5401, Japan). To calculate grip strength-related statistics, the maximum value of the dominant hand among the grip strength values of both hands measured three times was used. The maximum value among the grip strength values of both hands or one hand, measured three times from the 3rd year of the 7th year (2018), were used. Examination items included arm/hand/thumb defects, defects or fractures of fingers other than the thumb, hand paralysis, hand/wrist casts, and bandages. Survey items included hand/wrist within the last 3 months. Subjects for grip strength measurement were selected based on their history of surgery, arthritis, and carpal tunnel syndrome surgery; whether they could participate in subjective surveys; and experience or worsening of hand pain/throbbing/stiffness within the last 7 days. The grip strength of the hand was measured in a neutral position, with the index finger and handle at a 90° angle. The participants were measured three times at 60 s intervals on both hands. 

For the analysis, we divided handgrip strength into two categories. The cutoff values for muscles with low grip strength were <26 kg for men and <18 kg for women. These criteria were based on an Asian sarcopenia report [19].

### 2.5. Confounding Factor Assessment

The confounding factors were sex, age, monthly household income, education level, residential area, marital status, type of health insurance, private health insurance subscription, toothache in the past year, periodontal disease, brushing the previous day, and dental damage experience. Chewing problems, chewing complaints, speech problems, oral examinations for 1 year, use of dental clinics for 1 year, unsatisfactory dental treatment, smoking, drinking, physical activity, hypertension, diabetes, dyslipidemia, stroke, myocardial infarction, and angina pectoris were evaluated.

### 2.6. Statistical Analysis

The collected data were analyzed using SPSS 27.0 (IBM Corp., Armonk, NY, USA). All statistical analyses were performed using complex sampling methods. The number of remaining teeth, according to the characteristics of the participants, was analyzed using the Rao–Scott chi-square test. To overcome the confounding variables that were significant in the Rao–Scott chi-square test and to understand the relationship between grip strength and number of teeth, the relationship between grip strength and number of teeth was identified through multinomial logistic regression analysis. For all analyses, the statistical significance level was set at *p* < 0.05.

## 3. Results

### 3.1. Number of Remaining Teeth According to the General Characteristics of the Participant

The numbers of remaining teeth by sex in Korean adults were 85.6% with 20–28 teeth in females and 81.3% with 20–28 teeth in males. Most participants (94.7%) in the adult age group (19–64 years) were in the 20–28 group, and 52.3% of elderly people (≥65 years) were in the 20–28 teeth group. The number of remaining teeth according to monthly household income, including sex, age, education level, residence area, marital status, health insurance type, and private medical insurance subscription were all statistically significant (*p* < 0.001) (Table 1).

### 3.2. Number of Remaining Teeth According to the Oral Health Characteristics of the Participant

The results of identifying the number of remaining teeth according to the participants’ oral health characteristics are shown (Table 2). The number of remaining teeth was 20–28 in 79.6% cases with periodontal disease, significantly lower than 90.4% of adults without periodontal disease (*p* < 0.001). Adults with very uncomfortable chewing had fewer than 10 remaining teeth (30.8%), and those who complained of chewing discomfort had fewer than 10 teeth (17.5%). In addition, toothache in the past year, prevalence of periodontal disease, tooth brushing on the previous day, tooth damage, chewing problems, complaints of chewing discomfort, speaking problems, oral examination for one year, use of dental hospitals, unsatisfactory dental care, and all teeth showed statistically significant differences from the remaining number (*p* < 0.001).

### 3.3. Number of Remaining Teeth According to the Participant’s General Health

Looking at the number of remaining teeth according to the general health of Korean adults, 9.4% of smokers had less than 10 teeth, and 6.9% of non-smokers had less than 10 teeth. In addition, patients diagnosed with systemic diseases such as hypertension, physician diagnosis, stroke, myocardial infarction, angina pectoris, and diabetes were more likely to have fewer remaining teeth. Smoking, drinking, aerobic physical activity, and diagnosis of systemic diseases showed statistically significant differences (Table 3).

### 3.4. Relationship between the Participant’s Grip Strength and the Number of Remaining Teeth

The results of the multinomial logistic regression analysis identifying the relationship between grip strength and the number of remaining teeth are shown (Table 4). Among participants with fewer than 10 remaining teeth, the number of remaining teeth was 10–19 when the grip was normal, but there was no statistically significant difference (OR, 1.260; 95% CI, 0.924–1.719). However, when grip strength was normal, the number of remaining teeth was often 20–28, showing a statistically significant difference (OR, 1588; 95% CI 1.186–2.127). It was confirmed that the higher the probability of normal grip strength, the higher the number of residual teeth. This suggests that the greater the number of remaining teeth in Korean adults, the higher the level of muscle strength and the better the health.

The results were adjusted for: sex, age, household income, education level, residence area, marital status, health insurance type, private medical insurance, tooth pain, periodontal disease, tooth brushing, teeth damage, chewing problems, chewing discomfort, speaking problems, oral examination, dental use, unmet dental care needs, smoking, drinking, aerobic physical activity, hypertension, dyslipidemia, stroke, myocardial infarction, angina pectoris, and diabetes

## 4. Discussion

Our hypothesis was that hand strength is related to tooth loss. Therefore, the purpose of this study was to investigate the relationship between handgrip strength, which is a general strength indicator among Korean adults, and the number of remaining natural teeth. The results of this study showed that the greater the number of natural teeth remaining, the higher the probability of having a normal hand grip.

This result is similar to that of a previous study [3] that reported that the number of missing teeth is related to grip strength. In addition, it can be said that the lower the grip strength, the higher the frequency of periodontal disease [20]. This establishes an association between the number of natural teeth lost and handgrip strength. Study participants with weaker muscles have difficulty performing oral hygiene techniques (such as brushing) frequently and may lose teeth as a consequence. Conversely, poor dietary intake due to tooth loss and nutritional deficiencies associated with poor dietary intake can reduce handgrip strength, making frequent oral hygiene procedures difficult [21].

In addition, low grip strength is associated with sarcopenia, disability, and limited dexterity [22]. Previous studies reported an association between limited hand function, inadequate manual dexterity, and poor oral care skills [23]. Decreased grip strength can lead to periodontitis and tooth loss, due to the neglect of oral healthcare, including plaque removal [24]. In addition, if a reduction in grip strength negatively affects mastication, malnutrition can lead to a vicious cycle in which hand strength is further reduced. Previous studies examining the association between grip strength and oral health indicators have shown that low grip strength promotes early hand fatigue, resulting in reduced brushing time and the development of severe periodontal disease. This is because low grip strength increases the risk of periodontal disease [25]. This may lead to tooth loss.

Evidence for a link between the number of teeth and grip strength is still controversial. Chewing discomfort due to tooth loss can cause inadequate food intake, leading to undesirable food intake [26]. Eating habits are related to various chronic diseases [27]. As such, it can be seen that there is a significant correlation between low grip strength and health. Therefore, more research is needed to investigate the detailed mechanism linking the number of teeth and the force of the hand.

The study had some limitations. It was a cross-sectional study that confirmed the relationship between grip strength and tooth loss, and although the relationship was identified, it was difficult to explain a clear causal relationship. Because it was a cross-sectional survey, it was difficult to ascertain whether adults with weak grip strength had more tooth loss or whether the more teeth lost, the weaker the grip. Despite these limitations, the strength of this study was that potential risk factors, such as oral disease symptoms and dental visits related to oral health, were set as confounding factors to identify the relationship between grip strength and tooth loss. In addition, variables related to systemic diseases, which were not conducted in previous studies, were set as confounding factors. Based on the vast amount of data from the KNHANES, the relationship between grip strength and tooth loss was confirmed in a number of Korean adults, and the data of this study can be used as basic data for improving grip strength and preventing tooth loss in the future.

## 5. Conclusions

The results of this study showed a significant association between the number of teeth and grip strength in Korean adults. However, further studies are needed to clarify the causal relationship. In addition, it is necessary to investigate tooth loss and reduced grip strength due to oral diseases and to conduct cohort studies on reduced grip strength and systemic diseases due to tooth loss.

## Figures and Tables

**Table 1:** Prevalence of functional and non-functional dentitions.

Variable	The Number of Teeth			*p*-Value
	0–9	10–19	20–28	
Sex				<0.001
Male	9.4 (0.6)	9.3 (0.5)	81.3 (0.8)	
Female	6.3 (0.5)	8.2 (0.4)	85.6 (0.7)	
Age				<0.001
19–64	1.5 (0.2)	3.8 (0.3)	94.7 (0.3)	
≥65	25.0 (1.1)	22.7 (0.9)	52.3 (1.3)	
Household income				<0.001
Very low	22.7 (1.3)	18.7 (1.0)	58.6 (1.6)	
Low	8.4 (0.7)	11.7 (0.8)	79.9 (1.0)	
Normal	4.9 (0.5)	6.6 (0.6)	88.4 (0.8)	
High	2.6 (0.4)	5.0 (0.6)	92.4 (0.7)	
Very high	2.1 (0.4)	3.8 (0.5)	94.1 (0.6)	
Education level				<0.001
Elementary school	64.9 (2.2)	50.0 (2.2)	13.5 (0.6)	
Middle school	10.1 (1.1)	13.6 (1.1)	76.3 (1.5)	
Hihg school	3.5 (0.4)	6.0 (0.5)	90.5 (0.6)	
College	1.0 (0.2)	2.7 (0.3)	96.3 (0.4)	
Residence area				<0.001
City	6.1 (0.4)	7.7 (0.4)	86.2 (0.6)	
Country	14.6 (1.8)	13.7 (1.0)	71.8 (2.5)	
Marital status				<0.001
Married	8.8 (0.5)	10.0 (0.4)	81.2 (0.7)	
Single	0.6 (0.2)	1.3 (0.3)	98.1 (0.3)	
Health insurance type				<0.001
National Insurance (region)	7.7 (0.6)	10.6 (0.7)	81.7 (0.9)	
National Insurance (workplace)	6.4 (0.5)	7.4 (0.4)	86.2 (0.7)	
Medical benefit	21.2 (2.9)	15.7 (2.1)	63.1 (3.3)	
Private medical insurance				<0.001
Yes	3.0 (0.2)	6.0 (0.3)	90.9 (0.4)	
No	31.6 (2.1)	53.8 (2.0)	83.8 (0.6)	

**Table 2:** Number of teeth according to oral health characteristics.

Variable	Number of Teeth			*p*-Value
	0–9	10–19	20–28	
Tooth pain				<0.001
No	8.4 (0.5)	7.7 (0.4)	83.8 (0.7)	
Yes	5.4 (0.5)	10.7 (0.7)	83.9 (0.9)	
Periodontal disease				<0.001
No	3.6 (0.3)	6.1 (0.4)	90.4 (0.5)	
Yes	4.8 (0.4)	15.7 (0.8)	79.6 (0.9)	
Tooth brush yesterday				<0.001
No	60.7 (3.6)	10.4 (2.0)	29.0 (3.5)	
Yes	6.1 (0.3)	8.5 (0.4)	85.4 (0.6)	
Teeth damage				0.001
No	7.6 (0.4)	8.3 (0.4)	84.2 (0.6)	
Yes	5.5 (0.7)	11.0 (1.0)	83.5 (1.2)	
Chewing problems				<0.001
Very uncomfortable	30.8 (2.2)	28.4 (2.1)	40.9 (2.5)	
Inconvenient	14.2 (1.0)	16.8 (1.0)	68.9 (1.4)	
Commonly	7.0 (0.7)	10.3 (0.8)	82.7 (1.0)	
Comfortable	4.3 (0.4)	6.0 (0.6)	89.7 (0.7)	
Very comfortable	3.1 (0.3)	3.2 (0.4)	93.7 (0.5)	
Chewing discomfort				<0.001
No	4.3 (0.3)	5.6 (0.3)	90.1 (0.5)	
Yes	17.5 (1.0)	19.1 (0.9)	63.4 (1.3)	
Speaking problems				<0.001
Very uncomfortable	43.0 (4.0)	33.6 (3.8)	23.4 (3.7)	
Inconvenient	29.8 (2.0)	25.8 (1.7)	44.3 (2.2)	
Commonly	15.5 (1.4)	18.0 (1.4)	66.6 (1.8)	
Comfortable	7.1 (0.7)	12.3 (0.9)	80.5 (1.1)	
Very Comfortable	3.0 (0.3)	3.9 (0.3)	93.1 (0.4)	
Oral examination				<0.001
No	9.9 (0.5)	9.6 (0.4)	80.5 (0.8)	
Yes	2.7 (0.3)	6.8 (0.5)	90.5 (0.6)	
Dental use				<0.001
No	9.8 (0.6)	7.5 (0.4)	82.7 (0.8)	
Yes	5.4 (0.4)	9.4 (0.4)	85.2 (0.6)	
Unmet dental care needs				<0.001
Yes	6.6 (0.6)	10.4 (0.7)	83.0 (0.9)	
No	6.3 (0.4)	8.6 (0.4)	85.1 (0.7)	
No treatment need	11.0 (0.9)	6.2 (0.7)	82.8 (1.2)	

**Table 3:** Number of teeth according to general health characteristics.

Variable	Number of Teeth			*p*-Value
	0–9	10–19	20–28	
Smoking				0.009
No	6.9 (0.4)	8.6 (0.4)	84.5 (0.6)	
Yes	9.4 (1.0)	8.6 (0.7)	82.0 (1.2)	
Drinking				<0.001
No	10.1 (0.6)	10.5 (0.5)	79.4 (0.9)	
Yes	4.8 (0.4)	6.9 (0.4)	88.3 (0.6)	
Aerobic physical activity				<0.001
No	9.5 (0.5)	10.5 (0.5)	80.1 (0.8)	
Yes	4.1 (0.4)	6.2 (0.4)	89.7 (0.6)	
Hypertension				<0.001
No	4.4 (0.3)	5.8 (0.3)	89.8 (0.5)	
Yes	17.3 (0.9)	17.8 (0.9)	64.9 (1.2)	
Dyslipidemia				<0.001
No	6.7 (0.4)	7.3 (0.3)	86.0 (0.6)	
Yes	10.8 (0.8)	14.8 (0.9)	74.4 (1.2)	
Stroke				<0.001
No	6.9 (0.4)	8.3 (0.3)	84.8 (0.6)	
Yes	21.8 (2.9)	20.7 (2.7)	57.5 (3.3)	
Myocardial infarction				<0.001
No	7.0 (0.4)	8.6 (0.4)	84.4 (0.6)	
Yes	23.1 (4.5)	14.1 (3.5)	62.8 (4.7)	
Angina pectoris				<0.001
No	7.0 (0.4)	8.6 (0.4)	84.5 (0.6)	
Yes	18.0 (3.3)	12.0 (3.1)	70.0 (4.1)	
Diabetes				<0.001
No	6.4 (0.4)	7.6 (0.3)	86.0 (0.6)	
Yes	18.0 (1.3)	18.7 (1.4)	63.3 (1.7)	
Handgrip strength	23.52 ± 11.64	25.37 ± 11.47	30.42 ± 11.45	0.001
Low	23.4 (1.5)	17.6 (1.2)	59.0 (1.9)	<0.001
High	4.9 (0.3)	7.2 (0.3)	87.8 (0.5)	

**Table 4:** Multinomial logistic regression analysis of the relationship between grip strength and number of teeth.

Variable	Number of Teeth 10–19		Number of Teeth 20–28	
	Crude OR (95% CI)	Adjusted OR (95% CI)	Crude OR (95% CI)	Adjusted OR (95% CI)
Handgrip strength (HGS)				
Low	1.00	1.00	1.00	1.00
High	1.95 (1.57–2.42)	1.26 (0.92–1.72)	7.08 (5.88–8.53)	1.59 (1.19–2.13)

OR: adjusted odds ratio, 95% CI: 95% confidence interval.

## Data Availability

The data are available on the official KNHANES website (https://knhanes.kdca.go.kr/knhanes/sub03/sub03_02_05.do; accessed on 1 May 2023).
